# Surgical smoke exposure to patients and to healthcare workers in the operating room: a quantitative assessment

**DOI:** 10.1007/s00540-026-03672-5

**Published:** 2026-01-29

**Authors:** Shuse Matsuyama, Takashi Asai, Tomoyuki Saito, Tomoki Kiyono, Yasuhisa Okuda

**Affiliations:** 1 Department of Anesthesiology, Dokkyo Medical University Saitama Medical Center, 2-1-50, Minami-Koshigaya, Koshigaya, Saitama 343-8555 Japan; 2 Institute of Urban Innovation, Yokohama National University, 9-5 Tokiwadai, Hodogaya-Ku, , Yokohama City, 240-8501 Japan

**Keywords:** Operating room, Surgical smoke, Electrosurgical unit, Hazard

## Abstract

**Background:**

Surgical smoke produced by the use of an electrosurgical unit may have a negative effect to patients and healthcare workers in the operating room, but studies on this problem are insufficient.

**Methods:**

In 100 situations in which patients were undergoing four types of surgery under general anesthesia, the count of airborne particles (in 1.415 L) was measured using a particle counter to see possible differences at the patients and at the anesthesiologists, during different types of surgery, and at several locations.

**Results:**

The airborne particles during the use of an electrosurgical unit were significantly higher than before its use, both at the patient’s head (median: 56/L vs 3,514/L; 95%CI for the median difference: 769–7,699/L) and at the anesthesiologist’s position (230/L vs 6,907/L; 95%CI for the median difference: 2,945–13,196/L) (*p* < 0.0001). The airborne particles were significantly higher during cardiovascular surgery than during open abdominal surgery (median difference in increase: 8,439/L), significantly higher during open abdominal surgery than during head and neck surgery (2,654/L), and significantly higher during head and neck surgery than during laparoscopic surgery (1,442/L) (all *p* < 0.0001), and were high anywhere in the operating room and even outside the operating room door (always > 2,000/L).

**Conclusions:**

During the use of an electrosurgical unit, both the patients and healthcare workers are at increased risk of being exposed to high concentrations of airborne particles derived from surgical smoke.

## Introduction

Surgical smoke is an aerosolized byproduct generated when the targeted cells undergo a temperature rise by the use of an electrosurgical unit (such as a laser and electrocauterization instrument), leading to the rupture of the cell membrane and dispersion of intracellular particles into the surrounding air [1–3]. Surgical smoke contains approximately 150 different components, including benzene, harmful gases, viruses, and blood fragments [4–7].

While the long-term health effects of chronic exposure of healthcare workers to surgical smoke remain largely unknown, short-term exposure can be associated with respiratory complications (such as coughing and bronchial asthma [2, 8]) and other complications (such as ocular irritation, headache, and dizziness). In our experience, patients undergoing surgery while awake, such as those undergoing Cesarean section, would frequently report an unpleasant smell of smoke and have nausea and coughing.

The coronavirus disease 2019 (COVID-19) pandemic has also highlighted an increased risk of viral transmission to both patients and healthcare workers during aerosol-generating procedures [9, 10], and several studies have indicated that viruses or bacteria containing in surgical smoke could be transmitted to healthcare workers [1, 2, 6, 11].

Despite reports of these potential risks, only a small number of studies carried out quantitative assessments of surgical smoke exposure, and those are mainly on exposure to surgeons [3, 12]. Little is known about the exposure levels of surgical smoke to patients undergoing different types of surgery and other healthcare workers during different types of surgery in the operating room [13–17].

The objectives of our study were (1) to quantify the amount of surgical smoke exposure to the patients and the anesthesiologists; (2) to investigate whether the amount of surgical smoke differs between different types of surgery; and (3) to map the dispersion of surgical smoke within the operating room, during the use of an electrosurgical unit.

## Methods

The study protocol was submitted to the research ethics committee of Dokkyo Medical University Saitama Medical Center, which informed us that the study did not require a formal review, because the study was not a study on humans. Written informed consent from patients and from healthcare workers in the operating rooms was also waived. Nevertheless, we informed all the healthcare workers in advance of the purpose and the methods of the study, and informed them of the fact that they had the right to refuse us to carry out the study.

We studied 100 situations in which patients (whose tracheas were intubated) were undergoing the following types of surgery under general anesthesia: cardiovascular surgery, open abdominal surgery, head and neck surgery, and laparoscopic surgery. The cardiovascular surgery included coronary artery bypass grafting, aortic valve replacement, mitral valve repair, mitral valve replacement, total arch replacement, ascending aortic replacement, aortic root reconstruction and pericardial waffle procedure. Open abdominal surgery included bilateral salpingo-oophorectomy, total hysterectomy, total hysterectomy with bilateral salpingo-oophorectomy, abdominal myomectomy, nephrectomy, partial hepatectomy, pancreaticoduodenectomy, cholecystectomy and Y-graft replacement. Head and neck surgery included thyroid lobectomy, total thyroidectomy, segmental mandibular resection, superficial parotidectomy, neck dissection, partial glossectomy, total laryngectomy, submandibular gland malignant tumor resection with neck dissection, nasal cavity tumor resection, bilateral palatine tonsillectomy, and septoplasty. Laparoscopic surgery included ileocecal resection, appendectomy, colostomy, distal gastrectomy, ultra-low anterior resection, low anterior resection, distal pancreatectomy, cholecystectomy, nephrectomy, renal tumor resection, total prostatectomy using the robotic system, adnexectomy, and ovarian tumor resection.

All the operating rooms were maintained under positive pressure, with an air exchange rate of 20 to 30 cycles per hour and an unidirectional downward airflow system equipped with a high-efficiency particulate air (HEPA) filter. According to guidelines of the Healthcare Engineering Association of Japan (HEAS-02–2022), the cleanliness level of the operating rooms was classified as Class II (HEPA filter, minimum air change rate: 15 times per hour, positive pressure ventilation). The sizes of the operating rooms varied depending on the types of surgery being carried out: 214.8 m^3^ (8.9 m × 8.1 m × 3.0 m (height)) for cardiovascular surgery, 140.7 m^3^ (6.5 m × 7.2 m × 3.0 m) for open abdominal surgery, 132.6 m^3^ (6.0 m × 7.4 m × 3.0 m) for head and neck surgery, and 135.9 m^3^ (6.1 m × 7.4 m × 3.0 m) for laparoscopic surgery.

We used a handheld airborne particle counter (Airborne particle counter Handheld 3016, Lighthouse Worldwide Solutions, Fremont, USA), which can simultaneously count airborne particles of 6 different size ranges (0.3, 0.5, 1.0, 2.5, 5.0 and 10.0 μm), and displays a count for each particle size and total counts [9, 10]. The size resolution of the counter is less than or equal to 0.10. For example, the 0.3 μm particles measured by the counter refer to those with diameters ranging from 0.27 to 0.33 μm. The measurement efficiency of the counter is 50% at 0.3 μm and 100% at 0.45 μm or bigger (ISO 21501–4 compliant).^1^^*1^ The sampling flow rate is 2.83 L/min and each measurement takes 30 s, so the counter indicates the count of airborne particles in 1.415 L.

Airborne particles were counted at two locations: near the patient’s head and at the anesthesiologist’s position. The rationale for selecting the patient's head's position as a measurement location was to simulate a situation of an awake patient who was undergoing surgery in which an electrosurgical unit (without a built-in smoke evacuation system) was being used. The anesthesiologist’s position was defined as one meter distant from the patient’s head and one meter above the floor.

In each case, airborne particles were counted twice, before and during the use of an electrosurgical unit: once shortly after the start of surgery, and another within 1 min of the use of an electrosurgical unit. The intervals between the two measurements were between approximately 5 to 10 min. The count level is designed to decrease to less than 1 within 5 min,^2^^*1^ but if the count level did not decrease to less than 1 at the second and later measurement timings, the case was excluded from analysis. During these measurements, the door of the operating room was kept closed, and if it was opened, the case was excluded from analysis. In addition, if any of the healthcare workers coughed during the measurements, the case was excluded from analysis. Although we did not restrict the movements of healthcare workers in the operating room, they generally were standing still during these two measurements. In the situations where patients were undergoing laparoscopic surgery, airborne particles of the air emitted from laparoscopy machines were counted.

In another 5 situations in which 5 patients were undergoing cardiovascular surgery with median sternotomy, the spread of surgical smoke in the operating room was assessed. Airborne particles were counted at nine designated locations inside the operating room (at 2 m intervals) and outside (50 cm distant from the door and one meter above the floor) at a random order, approximately 1–15 min of the use of an electrosurgical unit. In none of the cases was an independent smoke evacuation equipment used in the operating room.

From the results, color mapping of the mean particle concentrations of the five situations, with different colors representing varying particle levels, was produced: particle concentrations between the nine measurement locations (cross marks in Fig. 5) were spatially interpolated using a piecewise cubic Bezier polynomial in the SciPy 1.16.0 Python library [18]. A piecewise Bezier curve is designed to pass through data points exactly [18], so the colors of Fig. 5 match exactly the input values (*i.e.*, the mean concentrations of the five situations) at the nine measurement points. The extrapolated data outside the measurement points were drawn by sequentially applying nearest-neighbor extrapolation and the piecewise cubic Bezier interpolation. Namely, the concentration values at the corners and at the midpoints of the room's edges (extrapolated values) were assumed to be equal to those at the nearest measurement points first, and then the values between the extrapolated and the measurement points were interpolated.

### Statistical analysis

All calculations were carried out with the count/1.415 L, and after calculation, the count/1.415 L was converted to the count/L, and the values were rounded to the nearest integer. The medians and the interquartile ranges (IQR) were calculated for the distribution of airborne particle counts, as the data were not normally distributed.

For each measurement, the counts of 6 different airborne particle sizes and total counts were analyzed. To calculate the proportion for each particle size in the 6 different airborne particle sizes, we ignored the possible count of particles smaller than 0.3 μm (which could not be counted by the device used).

The primary outcome measures were the counts of airborne particles before and during the use of an electrosurgical unit, and the counts of airborne particles between different surgeries. Wilcoxon signed-rank test was used to compare the total counts of airborne particles, before and during the use of an electrosurgical unit. Kruskal–Wallis test was used to compare the total counts of airborne particles between different surgeries. If this showed a significant difference, Mann–Whitney *U* test was used to compare the total counts of airborne particles between two types of surgery.

The secondary outcome measures were the degrees of exposure to surgical smoke. In our preliminary observation, we have confirmed that the biggest increase in the particle count in the operating room was detected during the use of an electrosurgical unit, and the second biggest increase during exhalations by humans. In our previous study, the median count of aerosol particles when measured 30 cm from the mouth of a subject lying in the operating room was 3/L (maximum: 51/L) (reported values were converted from /1.415L to /L), whereas the median count while a subject was strongly coughing was 51/L (interquartile range: 31–142/L; maximum: 332/L) [9].

Although the counts of aerosol particles produced by coughing and the counts of airborne particles produced by surgical smoke might not be equivalent, and although there is no standard for particulate matter in the operating room, we arbitrary judged that the concentrations of airborne particles was high, when the increase in the count of airborne particles during the use of an electrosurgical unit was 300/L (nearly the maximum level of counts during strong coughing [9]) or greater. We also judged that, the concentration of airborne particles was very high if the increase was 3,000/L or greater, and extremely high if the increase was 30,000/L or greater. In addition, the increase less than 300/L was categorized into the following: medium (the increase by 150/L or greater, but less than 300/L), minimum (by 75/L or greater, but less than 150/L), and negligible (by less than 75/L).

Chi-squared test was used to compare the incidences of a high concentration (300/L or greater) of airborne particles between the different types of surgery, and if this showed a significant difference, Fisher’s exact test was used to compare the incidences between two types of surgery. *P* < 0.05 was considered significant. 95%Confidence interval (CI) for the median differences between two different situations was calculated manually.

Power analysis was performed before the start of the study. For comparisons of the differences in the count of airborne particles before and during the use of an electrosurgical unit (paired data), 15 samples would be required to detect a large effect size (*d* = 0.8 defined by Cohen [19]), with a power of 0.8, and *P* = 0.05. For comparisons of the differences in the count of airborne particles between two types of surgery (unpaired data), 25 samples would be required to detect a large effect size (*d* = 0.8), with a power of 0.8, and *P* = 0.05. Therefore, we decided to study 25 samples for each type of surgery, or 100 samples in total.

## Results

The obtained data were used for analysis, without exclusions.

### Before and during the use of an electrosurgical unit

**Fig. 1:**
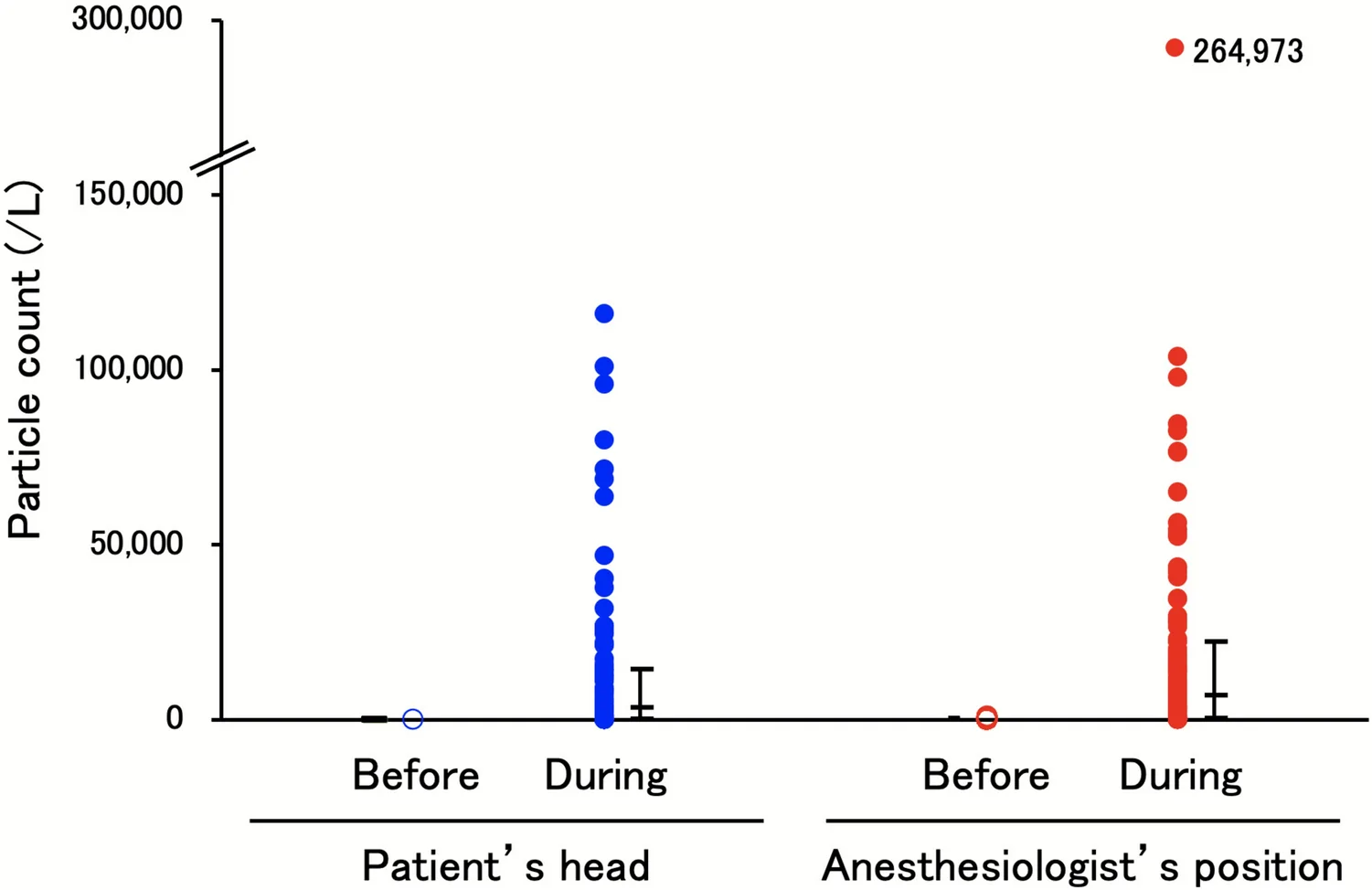
Individual values (and the medians and inter-quartile ranges) of the count of airborne particles before (open circles) and during (solid circles) the use of an electrosurgical unit, at the patient’s head (blue circles) and at the anesthesiologist’s position (red circles)

At the patient’s head, the median count of airborne particles was 56 (IQR: 13–126)/L before the use of an electrosurgical unit, and was 3,514 (117–14,288)/L during the use of an electrosurgical unit (Fig. 1). At the anesthesiologist’s position, the median count was 230 (134–370)/L before the use of an electrosurgical unit, and was 6,907 (396–22,353)/L during the use of an electrosurgical unit (Fig. 1).

The airborne particles during the use of an electrosurgical unit were significantly higher than before its use, both at the patient’s head (*p* < 0.0001; median difference 3,338 [95%CI for the median difference: 769–7,699]/L), and at the anesthesiologist’s position (*p* < 0.0001; median difference 6,430 [2,945–13,196]/L). There was no significant difference in the increase in the count of airborne particles during the use of an electrosurgical unit, at the patient’s head (3,337 (85 – 14,190)/L) and at the anesthesiologist’s position (6,430 (179 – 22,190)/L) (*p* = 0.07; median difference 77 [95%CI for the median difference: −1,596 – 1,833]/L.

### Types of surgery

**Fig. 2:**
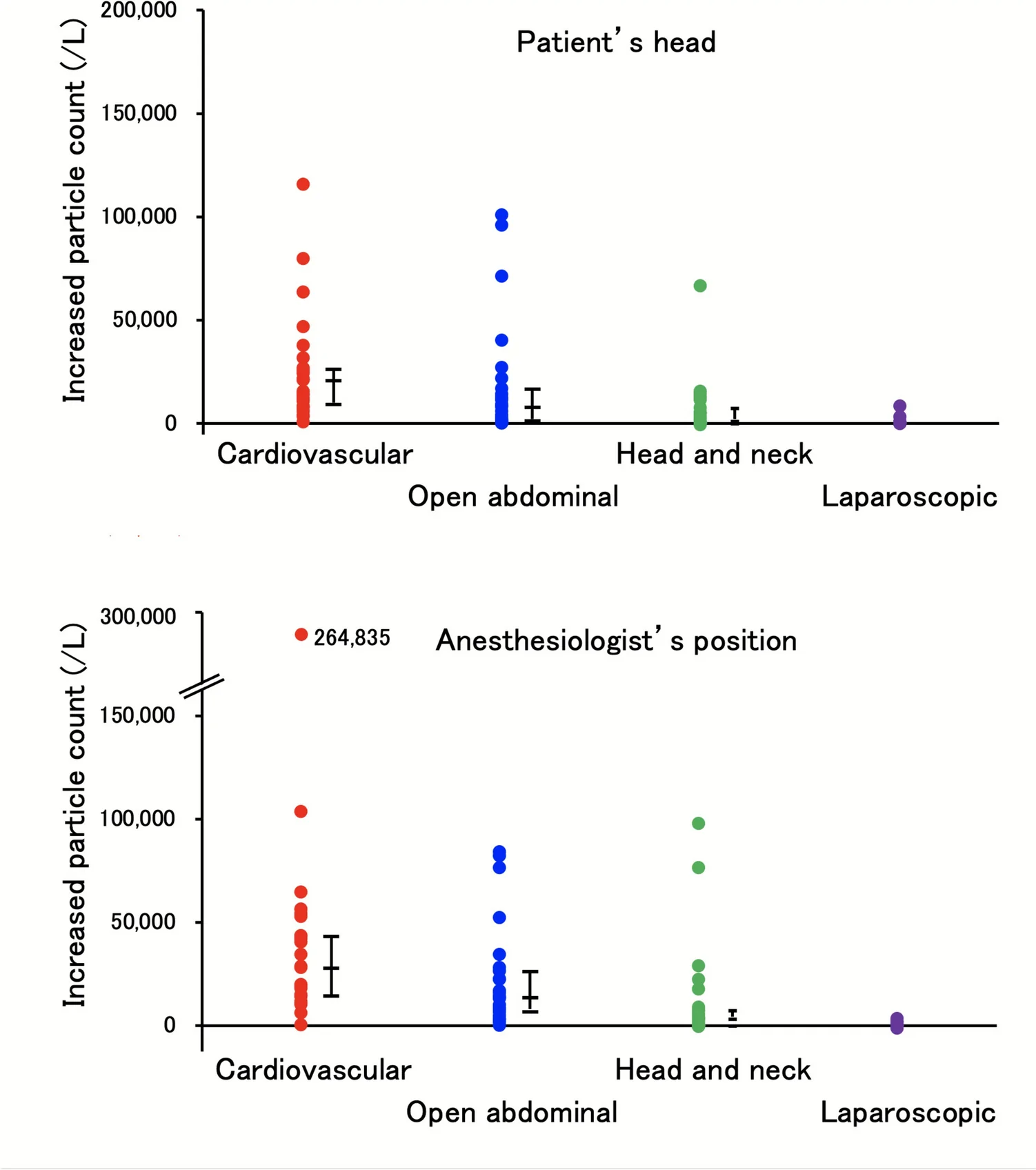
Individual values (and the medians and inter-quartile ranges) of the difference in the count of airborne particles (before and during the use of an electrosurgical unit) during cardiovascular surgery (red circles), during open abdominal surgery (blue circles), head and neck surgery (green circles) and laparoscopic surgery (purple circles), with the medians and inter-quartile ranges

There were significant differences in airborne particles between 4 different types of surgery (*p* < 0.0001 for both at the patient’s head and at the anesthesiologist’s position). Both at the patient’s head and anesthesiologist’s position, airborne particles were significantly higher during cardiovascular surgery than during open abdominal surgery, significantly higher during open abdominal surgery than during head and neck surgery, and significantly higher during head and neck surgery than during laparoscopic surgery (all *p* < 0.0001) (Fig. 2).

At the patient’s head, the median difference in the count of airborne particles was 8,439 [95%CI for the median difference: 1,899—16,790]/L between cardiovascular surgery and open abdominal surgery; 2,654 [142—9,053]/L between open abdominal surgery and head and neck surgery; and 1,442 [114—5,097]/L between head and neck surgery and laparoscopic surgery (Fig. 2a).

Similarly, at the anesthesiologist’s position, the median difference in airborne particles was 10,671 [796—23,717]/L between cardiovascular surgery and open abdominal surgery; 8,411 [3,020—14,227]/L between open abdominal surgery and head and neck surgery; and 3,081 [207—5,899]/L between head and neck surgery and laparoscopic surgery (Fig. 2b).

**Fig. 3:**
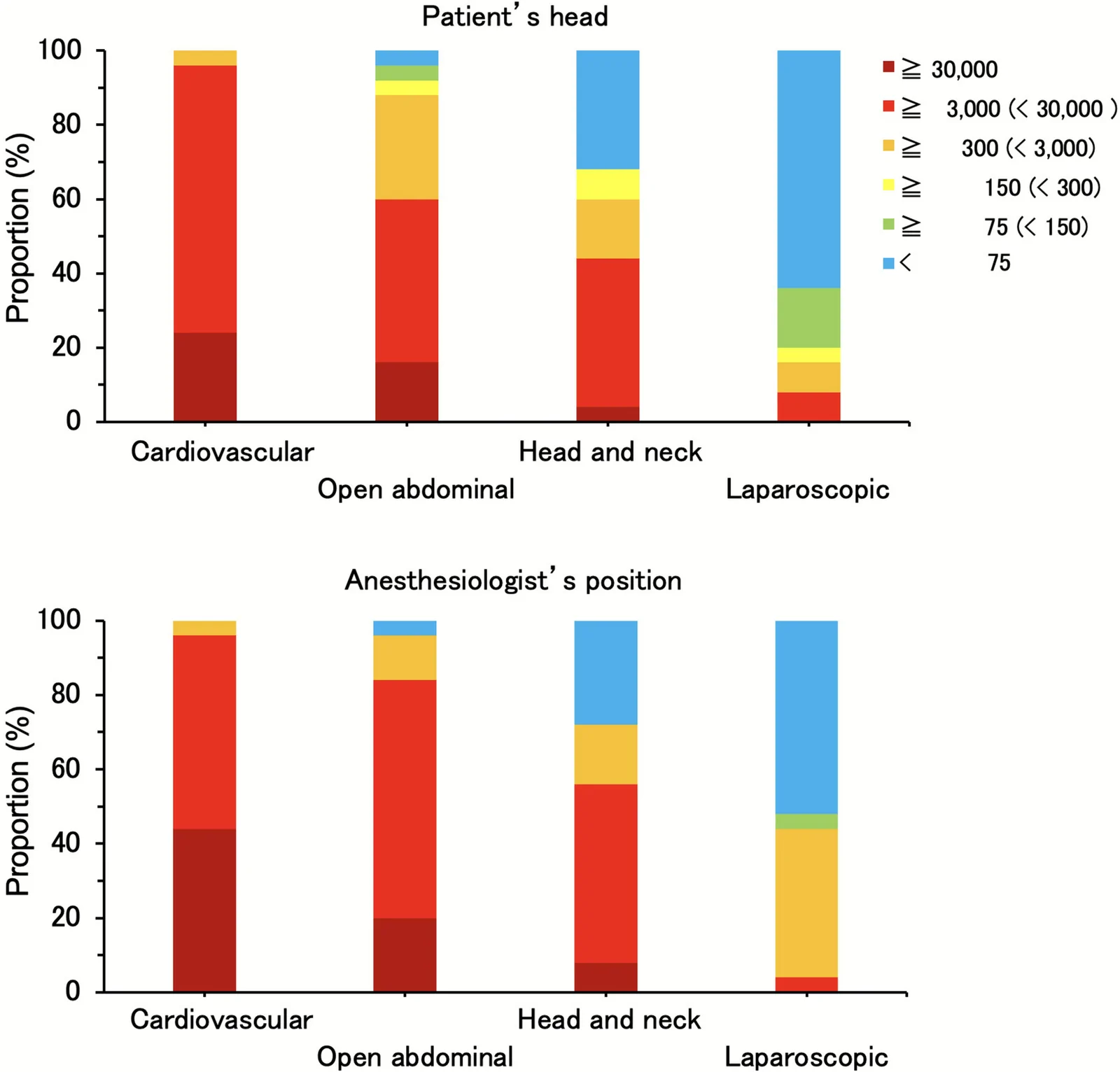
Proportions for the following 6 degrees of increase in the count of airborne particles during the use of an electrosurgical unit: extremely high (30,000 or greater); very high (3,000 or greater, but less than 30,000); high (300 or greater, but less than 3,000); medium (150 or greater, but less than 300); minimum (75 or greater, but less than 150); and negligible (less than 75)

The incidence of a high concentration of airborne particles (300 or greater) was significantly higher for cardiovascular surgery than for open abdominal surgery, significantly higher for open abdominal surgery than for head and neck surgery, and significantly higher for head and neck surgery than for laparoscopic surgery, both at the patient’s head and at the anesthesiologist’s position (*p* < 0.00001 to 0.05) (except for the fact that there was no significant difference between cardiovascular surgery and open abdominal surgery both at the patient’s head and at the anesthesiologist’s position) (Fig. 3). Although the count of airborne particles were lowest during laparoscopic surgery, the count exceeded 300 in 4 of 26 (15%) at the patient’s head and in 4 of 26 (15%) at the anesthesiologist’s position. The median count of airborne particles in the air emitted from the laparoscopy machine was 376 (IQR: 237—466)/L, with the maximum value of 558/L.

### Particle sizes

**Fig. 4:**
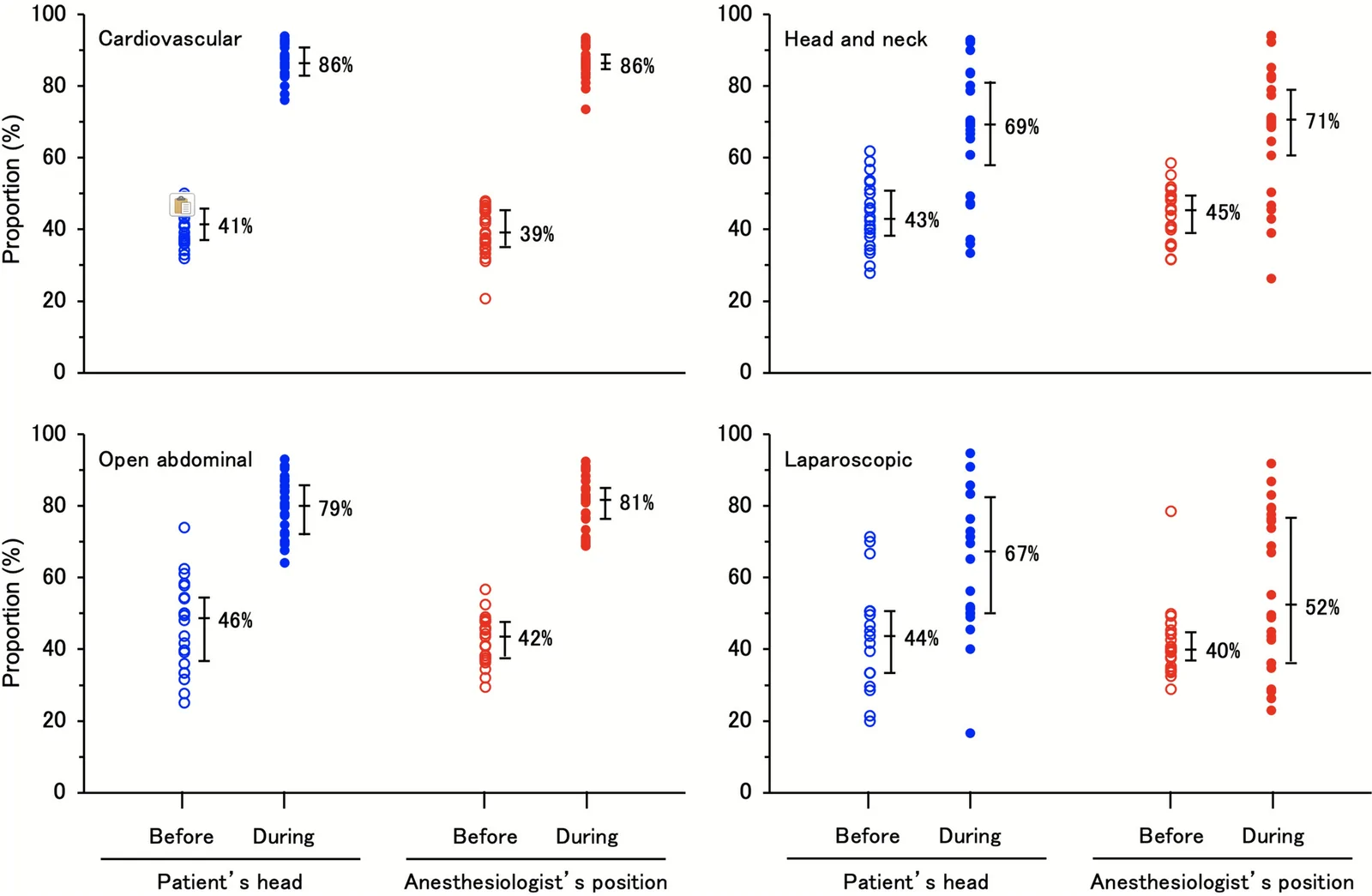
Individual values (with the medians and inter-quartile ranges) of the proportion of particles of the smallest size (0.3 μm) among 6 different airborne particle sizes (0.3, 0.5, 1.0, 2.5, 5.0 and 10.0 μm) counted, before and during the use of an electrosurgical unit, for four different types of surgery

During the use of an electrosurgical unit, all the particle sizes (0.3—10 μm) were detected, and the proportion of particles of the smallest size (0.3 μm) was the highest, for any type of surgery, but particularly high during cardiac surgery or during open abdominal surgery (Fig. 4).

### Spread of surgical smoke

Before the use of an electrosurgical unit on patients undergoing cardiovascular surgery, the counts of airborne particles were reasonably low at 9 designed points in the operating room, with the median counts of 199 (IQR: 131—236)/L, and outside the operating room (517 (480—879) L).

**Fig. 5:**
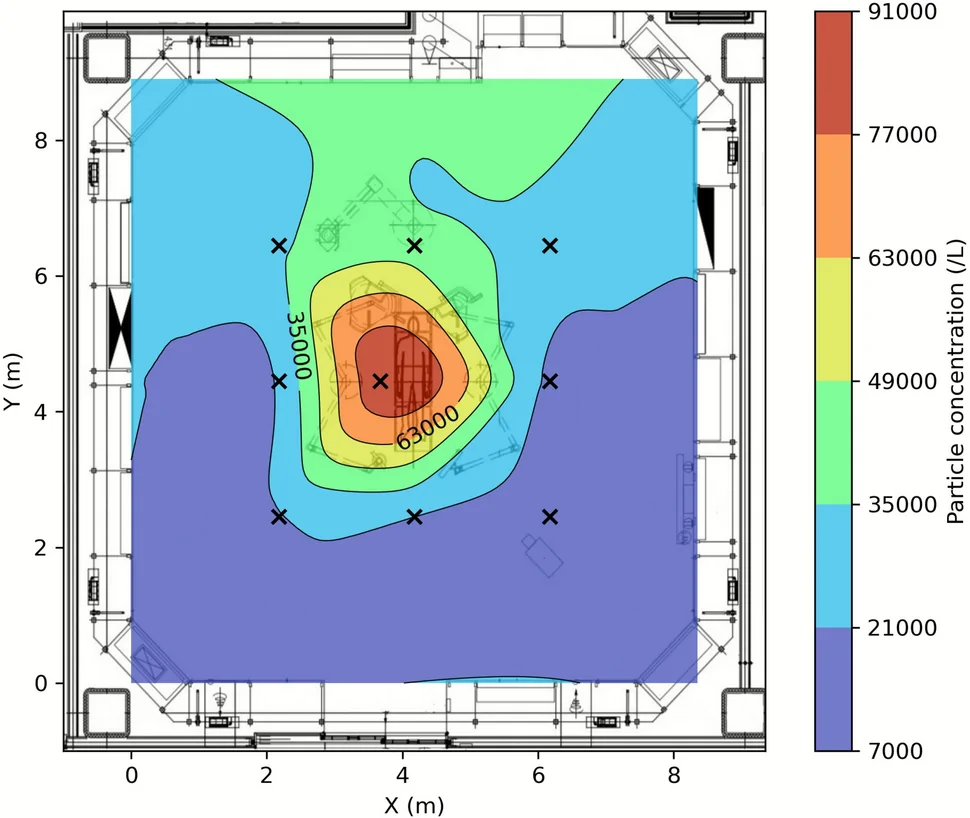
Color mapping (with different colors representing varying particle count levels) in an operating room during the use of an electrosurgical unit

During the use of an electrosurgical unit, the concentrations of airborne particles were judged very high (≧3,000) at all the 9 designed points in the operating room (Fig. 5), and were sometimes extremely high (≧30,000). The highest count was observed at the surgeon’s position. Even outside the operating room door, the counts of airborne particles always exceeded 2,000/L, and the concentrations of airborne particles were judged very high (≧3,000) in 4 of 5 situations.

## Discussion

We have shown that (1) extremely high amount of airborne particles would be detected during the use of an electrosurgical unit, both at the patient’s head and at the anesthesiologist’s position; (2) the amount of surgical smoke would be markedly different between different types of surgery; and that (3) surgical smoke would fill the entire space of the operating room, and high concentrations of airborne particles can be detected even outside the operating room.

In our study, the count of airborne particles during the use of an electrosurgical unit was high at both the patient’s head and at the anesthesiologist’s position, without a significant difference. In a previous study [11], in which the count of airborne particles was measured at the surgeon’s position and at the anesthesiologist’s position in an operating room, although not statistically compared, the count was high at both positions. In another study [14], which was published after the start of our study has shown that, although not statistically compared, the increase was higher at the surgeon’s position than at the anesthesiologist’s position. A study comparing the count of ultrafine particles at different locations in the operating room indicated that there was no much differences in the level at the surgeon’s position and at the nurse anesthetist’s position, when an electrosurgical unit with a built-in smoke evacuation system was used [15]. These results at anesthesiologist’s position are consistent with the results of our study.

Concentration of airborne particles was highest during cardiovascular surgery, followed by during open abdominal surgery, during head and neck surgery, and the lowest during laparoscopic surgery (Fig. 3). These marked differences between different types of surgery may support a theory that the larger the area of the incision by an electrosurgical unit, such as median sternotomy and open abdominal surgery, the greater the amount of airborne particles produced. Therefore, it is reasonable to conclude that healthcare workers in the operating room are at a high risk of being exposed to extremely or very high concentrations of airborne particles during cardiovascular surgery and during open abdominal surgery. In addition, the patient who is undergoing open abdominal surgery while awake (such as Cesarean section) would inhale high amount of surgical smoke.

As expected, the count of airborne particles in the operating room was much lower during laparoscopic surgery than during open surgery (Fig. 2), mainly because gas containing surgical smoke in the abdominal cavity is continuously removed by suction, and the removed air is released through a filtration unit (e.g. HEPA filter or ultra low penetration air (ULPA) filter) to the operating room. Nevertheless, the count sometimes exceeded 300 during laparoscopic surgery (Fig. 3). The reason for this is not clear, but one possible reason is that suctioned air (containing surgical smoke) from the abdominal cavity is released to the operating room, due to decreased efficacy of a filtration unit of the laparoscopy machine. Nevertheless, this is unlikely in our study, as only a trace amount of particles were detected from the air being released from the laparoscopy machine. A more likely possibility is that surgical smoke produced in the abdominal cavity might have gushed out through or around laparoscopic trocars, particularly when stopcocks are open to air [20, 21].

Spread of surgical smoke can be confirmed by the fact that, during the use of an electrosurgical unit, one can smell the smoke when one approach to the operating room, and when the door is opened, one would have a strong smell coming out from the operating room. These findings indicate that not only surgeons, but also patients, anesthesiologists, surgical nurses, and other healthcare workers in the operating room, are being exposed to high concentrations of airborne particles.

In our study, all the sizes of particles (0.3—10 μm) measured were detected during the use of an electrosurgical unit, the results of which are consistent with previous studies [22–24]. In those particles of different sized, the proportion of particles of the smallest (0.3 μm) was highest, and was approximately 80–85% during cardiac surgery and open abdominal surgery (despite the fact that the counter would fail to count 50% of particles of this size range) (Fig. 4). Although we could not measure the airborne particle sizes smaller than 0.3 μm because of the device used, the result indicates that, in a statistical sense of distribution, the proportion of particles smaller than 0.3 μm would be even much higher.

Because the airborne particles smaller than 3.0 µm can reach the alveoli, there are potential risk of causing acute and chronic inflammation, such as pulmonary congestion, interstitial pneumonia, bronchiolitis, and emphysematous changes [2, 24, 25].

Although our study has shown that the count of airborne particles increases markedly during the use of an electrosurgical unit, there is no clear indicator of how much increase can be regarded as clinically meaningful, because of the insufficient number of quantitative studies that assessed the relationship between the amount of surgical smoke and the incidence of possible adverse effects. In this study, we arbitrary defined that the concentrations of airborne particles was high when the increase was greater than 300/L, which was roughly 100 times higher than the count when a subject lying without coughing in the operating room (median of 3/L), and the maximum value when a subject made a strong coughing.

In our previous study [9], we reported that the count of aerosol particles in the operating room is less than 4/L, whereas the count outside the operating room, for example, in an “ordinary” room can be as high as approximately 7,000/L. Therefore, we assessed the *increase* in the count of aerosol particles from the non-use to the use of an electrosurgical unit, rather than the absolute count during the use of the electrosurgical unit.

In our another previous study, the count of aerosol particles increased up to 835/L when a subject was strongly coughing while high-flow nasal oxygenation was provided with the flow rate of 60L/min [10]. Therefore, although the differences in measurement methods should be considered, it would be reasonable to conclude that the *increase* in the count of airborne particles during the use of an electrosurgical unit can be regarded as high when the increase was greater than 300/L, very high if the increase was 3,000/L or greater, and extremely high if the increase was 30,000/L or greater.

A number of countries have established environmental standards for atmospheric airborne particles, and some guidelines recommend protective measures against surgical smoke exposure to healthcare workers[26, 27],^3^^*2^ but the overall implementation rate remains low [28, 29]. The results of our study have clearly shown that during the use of an electrosurgical unit, both the patients and healthcare workers are at an increased risk of being exposed to high concentrations of airborne particles, anywhere in the operating rooms. Wearing a surgical mask is not sufficient to prevent inhalation of small sized surgical smoke, volatile organic compounds [7, 30, 31], and thus more reliable protective measures, such as the use of a N95 mask and local smoke evacuation system [9, 10], would be required to protect both awake patients and healthcare workers working in the operating rooms.

### Limitations

Our study has several limitations. First, we did not assess possible influence of factors, such as the differences in the size of operating rooms, in the duration of the use of an electrosurgical unit, in the method of ventilation of the operating room (positively or negatively pressured), in the positions of ventilation outlet, in the frequency of door opening, the presence or absence of spontaneous breathing and in the number of healthcare workers present in the operating room or their movements, on the particle counts. Therefore, caution would be required to generalize the results of this study, particularly to the patients who are breathing spontaneously.

Second, although the extent of skin incision, types of electrosurgical unit used, and device output settings varied depending on the surgical procedure and on the individual surgeon, we did not assess if these differences might have influenced the results. Third, we did not compare the possible differences in the amount of airborne particles between different types of electrosurgical units, as a monopolar electrosurgical unit was used in the majority of patients included to the study. Therefore, the results may not be applicable to the cases where an electrosurgical unit, other than a monopolar unit, is being used.

Fourth, the airborne particle counter used in our study could not measure particles smaller than 0.3 µm, so the amount of particles smaller than 0.3 µm during the use of an electrosurgical unit is not known. Fifth, we did not continuously count airborne particles and did not assess how long the particle counts persist after stopping the use of an electrosurgical device, and thus the overall exposure level of each person for each surgery could not be determined. Finally, we did not study if surgical smoke contained infectious microorganisms, nor did we study acute and chronic effects of surgical smoke on patients and healthcare workers in operating rooms. Therefore, the actual influence is not known of airborne particles derived from surgical smoke to patients and healthcare workers in the operating room.

## Conclusions

We conclude that surgical smoke generated by an electrosurgical unit disperses not only around the patient’s head and the anesthesiologist’s position but across the operating room. Surgery requiring incision of wide areas, such as median sternotomy for cardiovascular surgery and open abdominal surgery, would generate significantly higher amount of air particles derived from surgical smoke.

## Data Availability

Data are available from the authors.

## Footnotes

1 *1 LIGHTHOUSE. Handheld particle counters. Last accessed September 21, 2025. https://www.golighthouse.com/zh/wp-content/uploads/2022/09/Handheld_Brochure_2016-3016-3016IAQ-5016_210124.pdf

2 *1 LIGHTHOUSE. Handheld particle counters. Last accessed September 21, 2025. https://www.golighthouse.com/zh/wp-content/uploads/2022/09/Handheld_Brochure_2016-3016-3016IAQ-5016_210124.pdf

3 *2 Anonymous (US Department of Labor Occupational Safety and Health Administration). Laser/electrosurgery plume. https://www.osha.gov/laser-electrosurgery-plume. Last accessed September 21, 2025.

## References

[1] Swerdlow BN. Surgical smoke and the anesthesia provider. J Anesth. 2020;34:575–84. 10.1007/s00540-020-02775-x 32296937

[2] Limchantra IV, Fong Y, Melstrom KA. Surgical smoke exposure in operating room personnel: a review. JAMA Surg. 2019;154:960–7. 10.1001/jamasurg.2019.2515 31433468

[3] Romano F, Gustén J, De Antonellis S, Joppolo CM. Electrosurgical smoke: ultrafine particle measurements and work environment quality in different operating theatres. Int J Environ Res Public Health. 2017;14:137. 10.3390/ijerph14020137 PMC5334691 28146089

[4] Pierce JS, Lacey SE, Lippert JF, Lopez R, Franke JE. Laser-generated air contaminants from medical laser applications: a state-of-the-science review of exposure characterization, health effects, and control. J Occup Environ Hyg. 2011;8:447–66. 10.1080/15459624.2011.585888 21726157

[5] Okoshi K, Hida K, Kinoshita K, Morishima T, Nagai Y, Tomizawa Y, et al. Measurement of particulate matter 2.5 in surgical smoke and its health hazards. Surg Today. 2022;52:1341–7. 10.1007/s00595-022-02473-z PMC8859931 35188600

[6] Kwak HD, Kim SH, Seo YS, Song KJ. Detecting hepatitis B virus in surgical smoke emitted during laparoscopic surgery. Occup Environ Med. 2016;73:857–63. 10.1136/oemed-2016-103724 27484956

[7] Kocher GJ, Sesia SB, Lopez-Hilfiker F, Schmid RA. Surgical smoke: still an underestimated health hazard in the operating theatre. Eur J Cardiothorac Surg. 2019;55:626–31. 10.1093/ejcts/ezy356 30388210

[8] Le Moual N, Varraso R, Zock JP, Henneberger P, Speizer FE, Kauffmann F, et al. Are operating room nurses at higher risk of severe persistent asthma? The nurses’ health study. Occup Environ Med. 2013;55:973–7. 10.1097/JOM.0b013e318297325b PMC3740047 23887704

[9] Fujishiro A, Asai T, Saito T, Okuda Y. Efficacy of an aerosol suction device Free-100M in removing aerosols produced by coughing to minimize COVID-19 infection. J Anesth. 2023;37:196–200. 10.1007/s00540-022-03144-6 PMC9713171 36449089

[10] Ugajin W, Asai T, Saito T, Okuda Y. Efficacy of a surgical mask during high-flow nasal oxygen therapy in preventing aerosol dispersion: a randomized controlled study. J Anesth. 2023;37:787–93. 10.1007/s00540-023-03237-w 37540302

[11] Rioux M, Garland A, Webster D, Reardon E. HPV-positive tonsillar cancer in two laser surgeons: case reports. J Otolaryngol Head Neck Surg. 2013;42:54. 10.1186/1916-0216-42-54 PMC3843579 24246045

[12] Brüske-Hohlfeld I, Preissler G, Jauch KW, Pitz M, Nowak D, Peters A, et al. Surgical smoke and ultrafine particles. J Occup Med Toxicol. 2008;3:31. 10.1186/1745-6673-3-31 PMC2621226 19055750

[13] Hofer V, Kriegel M. Exposure of operating room surgical staff to surgical smoke under different ventilation schemes. Indoor Air. 2022;32:e12947. 10.1111/ina.12947 34704629

[14] Rao RS, Sasiharan GM, Ganapathy S. Estimating air pollution in operation rooms generated from cautery, drills, and other equipment, utilizing a particulate counter and gas analyzer. Indian J Occup Environ Med. 2023;27:310–6. 10.4103/ijoem.ijoem_200_22 PMC10880823 38390480

[15] Liu N, Filipp N, Wood KB. The utility of local smoke evacuation in reducing surgical smoke exposure in spine surgery: a prospective self-controlled study. Spine J. 2020;20:166–73. 10.1016/j.spinee.2019.09.014 31542472

[16] Ragde SF, Jørgensen RB, Føreland S. Characterisation of exposure to ultrafine particles from surgical smoke by use of a fast mobility particle sizer. Ann Occup Hyg. 2016;60:860–74. 10.1093/annhyg/mew033 27255204

[17] Li CI, Pai JY, Chen CH. Characterization of smoke generated during the use of surgical knife in laparotomy surgeries. J Air Waste Manag Assoc. 2020;70:324–32. 10.1080/10962247.2020.1717675 31961784

[18] Virtanen P, Gommers R, Oliphant TE, Haberland M, Reddy T, Cournapeau D, et al. SciPy 1.0: fundamental algorithms for scientific computing in Python. Nat Methods. 2020;17:261–72. 10.1038/s41592-019-0686-2 PMC7056644 32015543

[19] Cohen J. Multiple regression and correlation analysis. In: Statistical power analysis for the behavioral sciences. 2nd ed. New York: Psychology Press; 1988. p. 19–74.

[20] Choi SH, Kwon TG, Chung SK, Kim TH. Surgical smoke may be a biohazard to surgeons performing laparoscopic surgery. Surg Endosc. 2014;28:2374–80. 10.1007/s00464-014-3472-3 24570016

[21] Robertson D, Sterke F, van Weteringen W, Arezzo A, Mintz Y, Technology Committee of the European Association for Endoscopic Surgery (EAES), et al. Escape of surgical smoke particles, comparing conventional and valveless trocar systems. Surg Endosc. 2023;37:8552–61. 10.1007/s00464-023-10444-9 PMC10615925 37794124

[22] Kondo A, Watanabe Y, Ishida M, Suzuki Y, Hirano S. Particle size distributions in surgical smoke generated by advanced energy devices: a meaningful perspective from an experimental study in the time of COVID-19. Ann Surg. 2021;273:e168–70. 10.1097/SLA.0000000000004807 33824251

[23] Born H, Ivey C. How should we safely handle surgical smoke? Laryngoscope. 2014;124:2213–5. 10.1002/lary.24624 24648147

[24] Zakka K, Erridge S, Chidambaram S, Beatty JW, Kynoch M, Kinross J, et al. Electrocautery, diathermy, and surgical energy devices: are surgical teams at risk during the COVID-19 pandemic? Ann Surg. 2020;272:e257–62. 10.1097/SLA.0000000000004112 PMC7467049 32541232

[25] Tomita Y, Mihashi S, Nagata K, Ueda S, Fujiki M, Hirano M, et al. Mutagenicity of smoke condensates induced by CO₂-laser irradiation and electrocauterization. Mutat Res. 1981;89:145–9. 7027028

[26] Anonymous (Association of perioperative registered nurses). Guideline summary: surgical smoke safety. AORN J. 2017;105:498–500. 10.1016/j.aorn.2017.02.008 28454615

[27] Williams K. Guidelines in practice: surgical smoke safety. AORN J. 2022;116:145–59. 10.1002/aorn.13745 35880929

[28] Steege AL, Boiano JM, Sweeney MH. Secondhand smoke in the operating room? Precautionary practices lacking for surgical smoke. Am J Ind Med. 2016;59:1020–31. 10.1002/ajim.22614 PMC5069165 27282626

[29] Spearman J, Tsavellas G, Nichols P. Current attitudes and practices towards diathermy smoke. Ann R Coll Surg Engl. 2007;89:162–5. 10.1308/003588407X155752 PMC1964566 17346413

[30] Monjezi M, Jamaati H. The effects of face mask specifications on work of breathing and particle filtration efficiency. Med Eng Phys. 2021;98:36–43. 10.1016/j.medengphy.2021.10.004 PMC8493656 34848036

[31] Breul S, Van Landuyt KL, Reichl FX, Högg C, Hoet P, Godderis L, et al. Filtration efficiency of surgical and FFP3 masks against composite dust. Eur J Oral Sci. 2020;128:233–40. 10.1111/eos.12697 32378254

